# Noninvasive quantification of oxygen saturation in the portal and hepatic veins in healthy mice and those with colorectal liver metastases using QSM MRI

**DOI:** 10.1002/mrm.27571

**Published:** 2018-11-19

**Authors:** Eoin Finnerty, Rajiv Ramasawmy, James O’Callaghan, John J. Connell, Mark Lythgoe, Karin Shmueli, David L. Thomas, Simon Walker‐Samuel

**Affiliations:** ^1^ Department of Medical Physics and Biomedical Engineering University College London London United Kingdom; ^2^ Department of Medicine UCL Institute of Child Health, University College London London United Kingdom; ^3^ Institute of Neurology University College London London United Kingdom; ^4^ Department of Medicine University College London London United Kingdom

**Keywords:** cancer, hepatic venous oxygen saturation, liver, QSM

## Abstract

**Purpose:**

This preclinical study investigated the use of QSM MRI to noninvasively measure venous oxygen saturation (SvO2) in the hepatic and portal veins.

**Methods:**

QSM data were acquired from a cohort of healthy mice (n = 10) on a 9.4 Tesla MRI scanner under normoxic and hyperoxic conditions. Susceptibility was measured in the portal and hepatic veins and used to calculate SvO2 in each vessel under each condition. Blood was extracted from the inferior vena cava of 3 of the mice under each condition, and SvO2 was measured with a blood gas analyzer for comparison. QSM data were also acquired from a cohort of mice bearing liver tumors under normoxic conditions. Susceptibility was measured, and SvO2 calculated in the portal and hepatic veins and compared to the healthy mice. Statistical significance was assessed using a Wilcoxon matched‐pairs signed rank test (normoxic vs. hyperoxic) or a Mann‐Whitney test (healthy vs. tumor bearing).

**Results:**

SvO2 calculated from QSM measurements in healthy mice under hyperoxia showed significant increases of 15% in the portal vein (*P* < 0.05) and 21% in the hepatic vein (*P *< 0.01) versus normoxia. These values agreed with inferior vena cava measurements from the blood gas analyzer (26% increase). SvO2 in the hepatic vein was significantly lower in the colorectal liver metastases cohort (30% ± 11%) than the healthy mice (53% ± 17%) (*P *< 0.05); differences in the portal vein were not significant.

**Conclusion:**

QSM is a feasible tool for noninvasively measuring SvO2 in the liver and can detect differences due to increased oxygen consumption in livers bearing colorectal metastases.

## 1 INTRODUCTION

The measurement of blood susceptibility and venous oxygen saturation (SvO_2_) using QSM MRI has been the focus of several studies in recent years.^1^, ^2^, ^3^, ^4^ It has been shown in both animal models^3^ and humans^2^, ^5^, ^6^ that QSM can quantify changes in blood deoxyhaemoglobin content brought about by a hyperoxic gas challenge.^2^, ^3^ This measurement can be used together with arterial spin labeling measures of cerebral blood flow to estimate the cerebral metabolic rate of oxygen consumption^4^ and can even quantify regional venous oxygenation in the brain.^1^ To date however, QSM‐based SvO_2 _research has been carried out exclusively in the cerebral vasculature. In this study, we aimed to explore whether this technique can be extended to noninvasively quantify SvO_2_ in the liver.

In the liver, the portal vein transports blood from the gut and mesentery into the hepatic portal system of the liver. From there, blood passes through the liver parenchyma into the hepatic veins, which empty into the inferior vena cava (IVC). We can define SvO_2_ measured in the portal vein as *SpvO_2_* and that measured in the hepatic vein as *ShvO_2_*.

ShvO_2_ is an indicator of the oxygen supply to demand ratio in the liver^7^; currently it can only be measured invasively via catheterization. Several studies have shown the benefit of ShvO_2_ measurements,^8^, ^9^, ^10^ particularly in animals and patients that have undergone surgical procedures.^11^ For example, it was found that after Fontan operations (a palliative procedure performed on children), monitoring ShvO_2_ in the immediate postoperative period could predict the occurrence and severity of subsequent acute liver dysfunction.^12^ Likewise, it was shown that ShvO_2_ could be used to gauge the regeneration status of the remnant portion of the liver in rats that had undergone partial hepatectomy.^11^, ^13^

Given the demonstrated benefits of invasive ShvO_2_ measurements, we sought to investigate the potential of QSM to measure ShvO_2_ noninvasively in mice. A hyperoxic gas challenge was administered to a cohort of healthy mice, and ShvO_2_ and SpvO_2_ were calculated from susceptibility measured in branches of the hepatic and portal veins under normoxic and hyperoxic conditions. ShvO_2_ estimates were compared to independent measurements of IVC blood samples under the same conditions made using a blood gas analyzer. Lastly, ShvO_2_ and SpvO_2_ were calculated under normoxic conditions in mice that had been inoculated with colorectal liver metastases, and values were compared to the healthy cohort. It was hypothesized that changes in blood oxygen saturation in response to the gas challenge in the healthy animals will be systemic and thus will manifest in both portal and hepatic venous blood. Conversely, given the increased metabolic burden that cancer places on host tissue, differences in venous blood oxygen saturation between healthy and tumor‐bearing mice will manifest in the hepatic vein only, because the disease is confined to the liver and thus is not systemic.

## 2 METHODS

### 2.1 Animal preparation

All animal studies were performed in accordance with the UK Home Office Animals Science Procedures Act (1986) and UK National Cancer Research Institute guidelines. CD1 mice (n = 10, female 8–12 weeks) were anaesthetized using 4% isoflurane in 100% O_2_. Respiratory rate was constantly monitored using a pressure pad (SA Instruments, Stony Brook, NY) and maintained at ~40 to 80 breaths per min by varying isoflurane concentration between 1.5% and 3%. Body temperature was maintained at 37.5 ± 0.5^o^C using a warm‐water circulation system. Mice were selected and scanned in random order from each cohort.

Colorectal liver metastases were induced in severe combined immunodeficiency (CD1 background) mice (n = 10), which were inoculated with 1 × 10^6^ SW1222 colorectal liver metastases cells via intrasplenic injection,^14^ followed immediately by a splenectomy. Mice were scanned at 19 days postsurgery. All animals survived to the end of the study and gave analyzable data that is included in the results.

Gases were administered through a nose cone at a rate of 0.5 L/min. In all cases, MR images were acquired under normoxic conditions while the subject was administered medical air (21% O_2_/79% nitrogen). Hyperoxia was induced in the healthy cohort via the administration of 100% O_2_. Ten minutes were allowed between gases to allow the animals to acclimatize before QSM imaging.

### 2.2 MRI data acquisition

All subjects were imaged on a 9.4 Tesla (T) MRI system (Agilent Technologies, Santa Clara, CA) with a 39‐mm‐diameter transmit/receive birdcage coil (Rapid Biomed, Rimpar, Germany). Susceptibility data were acquired using velocity‐compensated^15^ 2D single echo, T_2_*‐weighted gradient echo acquisitions. Scan parameters included TR = 1000 ms; TE = 4 ms; flip angle = 70^o^; voxel size = 200 × 200 µm^2^; readout acquisition bandwidth = 50 kHz; number of averages = 8; and slice thickness = 200 µm^2^ with no slice gap.

A TE of 4 ms was chosen because it matches the T_2_* of the liver tissue at 9.4T.^16^ The FOV in each case was adjusted to ensure full coverage of the liver and water reference (see below), and matrix size was adjusted such that voxel size was maintained and the data were spatially isotropic. The number of slices varied from 60 to 80 to accommodate full coverage of the liver. Mice bearing tumors were at an advanced stage of disease and did not tolerate Anesthesia well. Hence, to decrease scan time and ensure that all mice survived the imaging protocol, all acquisitions were single echo, and signal averages were reduced to 4 when acquiring data from the tumor‐bearing mouse.

All acquisitions were first‐order flow (i.e., velocity) compensated in all cardinal directions, which required modification of the acquisition sequence. Briefly, moving isochromats accumulate extra phase with respect to static spins as they move through spatial localization gradients. The acquisition sequence was modified to include extra lobes in each gradient waveform to ensure that static isochromats and those moving at a constant velocity would all rephase at the desired echo time. The amplitude and duration of the extra lobes were calculated based on those calculated by the standard acquisition sequence using the approach taken by Bernstein et al.^17^

All MRI acquisitions were respiratory‐gated. This was done by monitoring the animals’ breathing rate in the scanner with a pressure sensitive respiratory monitor (SA Instruments, Stony Brook, NY). To avoid respiratory‐related motion artefacts, imaging data were only acquired during a flat region of the respiratory cycle (i.e., between breaths). Total scan time was 20 to 40 mins, depending on respiration rate. As the sequences were respiratory‐gated, the *average* TR was dependent on the respiratory rate of each subject; however, given the average respiratory rate of ~60 breaths per min, it is estimated that the average TR closely reflects the prescribed TR of 1000 ms. It was found that this usually allowed the acquisition of data from every slice within a single breath, thus further reducing respiratory artefact.

### 2.3 Water reference calibration

**Figure 1:**
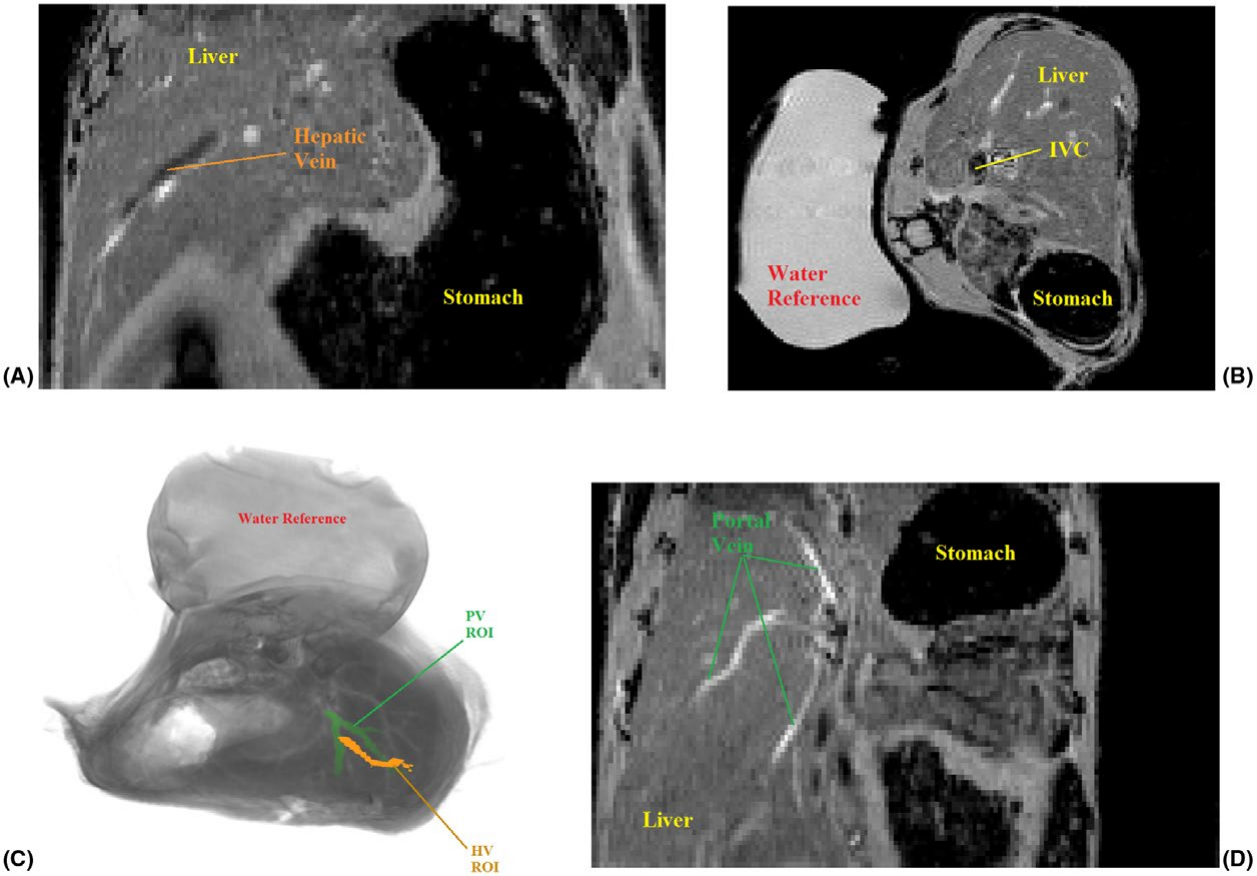
T_2_*‐weighted magnitude images showing example ROIs. (A) Image showing large branch of HV (coronal orientation) including liver and stomach. (B) Image showing water reference in situ, including IVC (axial orientation), stomach, and liver tissue. (C) 3D rendering of magnitude image displaying PV and HV ROIs and water reference in situ. (D) Image showing large branches of PV (coronal orientation) including liver and stomach. Abbreviations: HV, hepatic vein; IVC, inferior vena cava; PV, portal vein; ROI, region of interest

For quantification of susceptibility from QSM data, a reference material must be used for calibration.^18^ In the brain, this is usually CSF within ventricles,^19^ but in the liver no comparable material is available. We therefore included a sample of distilled water in the scanner with each subject (Figure 1). Briefly, a thin, cylindrical, nitrile membrane (~6 cm length, ~2 cm diameter) was filled with distilled water and sealed, with care taken to ensure that no air became trapped in the process. This was placed beneath each mouse in the animal holder before scanning commenced. All susceptibility values are quoted with respect to the water reference.

### 2.4 Image processing and analysis

Susceptibility maps were calculated from raw phase data. A binary mask was manually segmented around the entire liver in each magnitude image using ITK‐SNAP^20^ and included a large portion of the water reference. Phase unwrapping and background field suppression were performed using a Laplacian‐based Sophisticated Harmonic Artefact Reduction for Phase algorithm (Truncated Singular Value Decomposition threshold = 0.04, mask erode = 2–3 voxels).^21^

Susceptibility inversion was carried out using the thresholded k‐space division algorithm.^22^ The threshold of the kernel was set to ±0.2 such that absolute values outside of this range were set to the threshold value, with the appropriate sign depending on the position of the voxel within the kernel. This value was found empirically to offer the best balance between image fidelity and noise artefact. A correction factor of 1.26 (i.e., 1/0.786) was included in the deconvolution operation in the algorithm to correct for the underestimation inherent to this technique.^21^ All postprocessing was performed in MatLab version 2015b (MathWorks, Natick, MA).

Experimenters were not blinded to the treatment group at either acquisition or analysis. Regions of interest (ROIs) were manually segmented on each magnitude image using ITK‐SNAP^20^ and corresponded to large branches of the hepatic vein and the portal vein. To avoid partial volume effects, only voxels in the highest 20th percentile of susceptibility values per ROI were accepted.^1^

### 2.5 Calculating SvO_2_

The susceptibility difference between blood and water can be related to SvO_2_ by the following:

(1) $$\Delta \chi_{\text{blood - water}}=\Delta \chi_{\text{do}}\cdot \text{Hct}\cdot \text{(1 -}\;{\text{SvO}}_{2}),$$

where Δχ_do _= 2.26 parts per million (ppm) is the difference in susceptibility between fully oxygenated and deoxygenated blood at 100% hematocrit (Hct),^23^ and Hct is the fraction of blood composed of erythrocytes, assumed here to be 0.4.^3^ All calculations performed in this study used the water sample as the reference point.

### 2.6 Blood gas measurement

As an independent measurement for comparison with QSM estimates, blood gases were measured invasively in 3 mice randomly selected from the healthy cohort. The collection of murine hepatic blood is technically very challenging due to the size of the vessels under examination and the small volume of blood that can be sampled in a mouse. On this basis, extraction of blood from the portal vein was not possible; comparisons were instead drawn from measurements in the IVC, which was assumed to be representative of measurements in branches of the hepatic vein as they drain into the IVC.

Mice were anesthetized as described above, and a 1 mL syringe with 27 G needle was used to extract blood from a portion of the IVC within the liver under ultrasound guidance (Vevo 770, VisualSonics, Netherlands). The procedure was carried out under normoxic and hyperoxic conditions for each mouse as per MRI experiments. Ten minutes were allowed following a change in administered gas to allow the animal to acclimatize before sampling. Samples were transferred from the syringe to a 2 µL heparinized glass tube, and then to the blood gas analyzer (RAPIDLab 348EX blood gas system; Siemens, Frimley, UK).

### 2.7 Statistical analysis

A Wilcoxon matched‐pairs signed rank test was used to assess the statistical significance of the differences between normoxic and hyperoxic conditions, and a Mann‐Whitney test was used to compare differences between the healthy and tumor‐bearing mice. A difference was considered statistically significant for when *P* < 0.05.

## 3 RESULTS

### 3.1 QSM measurements of blood saturation in healthy hepatic vasculature

**Figure 2:**
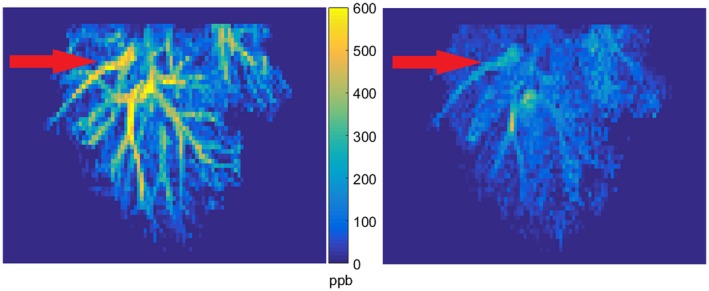
Maximum intensity projections of processed QSM data from a 2.2 mm segment of a representative mouse liver under normoxic (left) and hyperoxic (right) conditions. Large branches of the HV are clearly visible in each image (red arrows). Vessels are brighter with respect to the liver tissue (by approximately 500 ppb) in the normoxic image compared with the hyperoxic image, indicating a more paramagnetic susceptibility

The susceptibility of the blood in the large branches of mouse liver vasculature became more diamagnetic in response to the administration of O_2_. This is illustrated in Figure 2, which shows maximum intensity projections from susceptibility data acquired in a healthy mouse liver (11 slices, 2.2 mm segment), calculated from data acquired under normoxic and hyperoxic conditions. The blood vessels in the normoxic image are more prominent with respect to the liver tissue due to the increased presence of deoxyhemoglobin.

Magnetic susceptibility decreased significantly in the portal and hepatic veins of the healthy animals in response to hyperoxia. Mean susceptibility of the blood in the portal vein decreased from 380 ± 130 ppb under normoxia to 250 ± 160 ppb under hyperoxia (*P* < 0.05 n = 10). Similarly, susceptibility decreased from 430 ± 160 ppb to 230 ± 80 ppb in the hepatic vein under normoxic and hyperoxic conditions, respectively (*P* < 0.01, n = 10).

**Figure 3:**
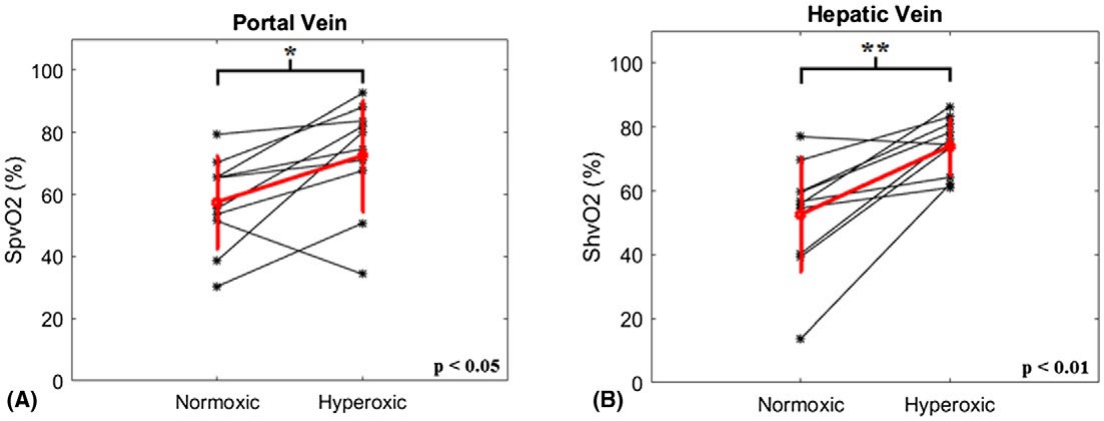
Change in venous oxygen saturation in (A) the PV (SpvO_2_) and (B) the HV (ShvO_2_). A statistically significant increase in oxygen saturation was measured in response to hyperoxia in both vessels (*P < 0.05, **P < 0.01).

Consequently, venous oxygen saturation increased significantly in both the portal and hepatic veins of the healthy animals in response to the administration of pure O_2_ (Figure 3). Portal venous oxygen saturation (SpvO_2_) (Figure 3A) increased by 15% from 58% ± 15% during air‐breathing to 73% ± 18% during hyperoxia (*P* < 0.05, n = 10), whereas in the hepatic vein (Figure 3B) ShvO_2_ increased by 21% from 53% ± 18% to 74% ± 9% (*P* < 0.01).

### 3.2 Invasive measurements of hepatic vein blood oxygen saturation

**Figure 4:**
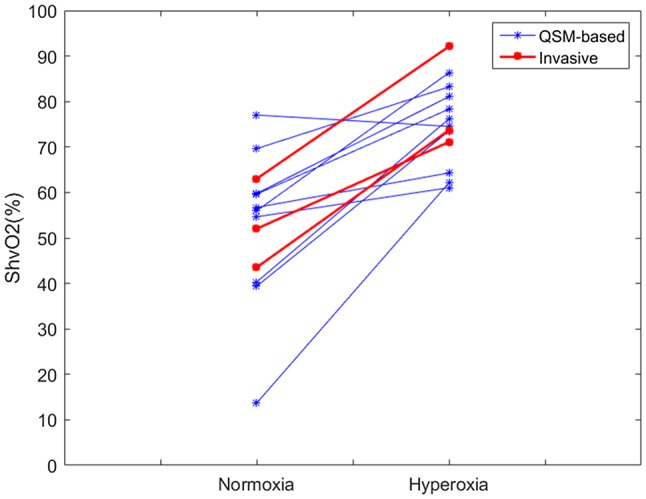
The change in venous oxygen saturation in the HV from noninvasive measurements with QSM and invasive measurements from the IVC with a blood gas analyzer. There is good agreement between the 2 datasets

There was good agreement between the measurements of ShvO_2_ acquired on the blood gas analyzer and those calculated from QSM measurements (Figure 4). The invasive measurement showed a mean increase of 26% in blood oxygenation, from 53% ± 10% to 79% ± 11%. This increase was not statistically significant. The invasive and noninvasive results were compared under normoxic and hyperoxic conditions using a Mann‐Whitney test. No significant differences were found between the invasive and noninvasive results under each condition.

### 3.3 QSM measurements of blood saturation in CRLM hepatic vasculature

**Figure 5:**
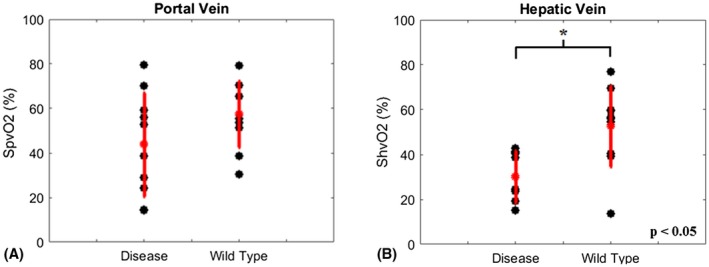
Measurements of venous blood oxygen saturation in mice with colorectal liver metastases and healthy mice. SvO_2_ values were calculated from susceptibility measurements in the PV (A) and the HV (B). Measurements in the HV of the mice with tumors contained significantly less oxygen than the healthy cohort

The graphs in Figure 5 show the venous blood oxygen saturation measured under normoxic conditions in the portal (A) and hepatic (B) veins of the mice with tumors and the healthy cohort. Mean oxygen saturation in the portal vein was 44% ± 23% and 58% ± 15% for the disease and healthy animals, respectively, and was not significantly different between the cohorts. Hepatic venous oxygen saturation (ShvO_2_) in the tumor‐bearing mice was significantly lower than that of the healthy cohort (*P* < 0.05). Mean values were 30% ± 11% and 52% ± 18% for the disease and healthy animals, respectively.

## 4 DISCUSSION

QSM is an exciting field that has given researchers a novel way to explore tissue composition and microstructure. Initial applications focused on quantifying the changes in tissue iron content that are indicative of neurological disorders, but the field has grown rapidly to give rise to a diverse range of clinically relevant applications. The work described here is the first attempt at using QSM to measure the susceptibility, and subsequently calculate the oxygen saturation, of blood in the major hepatic vessels.

There are a number of existing MR techniques that allow the noninvasive quantification of blood oxygenation, for example, R_2_*,^24^ T_2_,^25^ or SWI^26^; however, QSM offers a number of advantages over other MR methods. For instance, QSM measures bulk susceptibility, which is directly dependent on the iron content of the tissue, whereas R_2_* is affected by microscopic field inhomogeneities caused by the presence of iron and also includes pure R_2_ effects. As such, it has been shown previously that there is a stronger correlation between tissue iron content and susceptibility than with R_2_*.^22^ Secondly, R_2_* maps may be affected by intravoxel spin dephasing in the vicinity of air–tissue interfaces,^27^ an issue that is particularly prevalent when performing abdominal imaging. Thirdly, QSM has overcome the nonlocal phase contrast as well as the tissue geometry and orientation dependence that is known to affect SWI.^28^ As such, it is possible to differentiate between positive and negative susceptibility on QSM images, whereas the appearance of blood vessels in SW images can depend on the orientation of the vessel to the B_0_ field.

The aim of this study was to assess the ability of QSM to measure changes in venous blood oxygenation in the major hepatic vessels of mice in response to a hyperoxic gas challenge. In a previous study, QSM measurements of SvO_2_ were performed in the cerebral vasculature of mice and reported changes from ~88% to ~99% in response to hyperoxia.^3^ Absolute ShvO_2_ values calculated in the current study were lower (~52%, normoxia; ~74%, hyperoxia). Although there is little in the literature concerning ShvO_2_ in preclinical experiments, the values measured here are broadly in line with similar experiments in rats (~60% during normoxia).^11^, ^13^ Similarly, previous reports in the literature of the response of portal venous blood in rats to hyperoxia describe an increase from ~53% under normoxia to ~93% under hyperoxia in healthy control animals.^29^ It is therefore encouraging that this normoxic SpvO_2_ value is comparable to the measurement made here. Indeed, while the increase under induced hyperoxia was greater than we observed, this could be due to differences between experimental protocols, the efficiency of oxygen delivery, or differences between species. Moreover, a good agreement was found between QSM‐based and invasive measurements of hepatic venous oxygen saturation during both normoxia and hyperoxia. The difference brought about by the gas challenge as measured invasively was not statistically significant, which could be due to the small size of the cohort.

In accordance with our hypothesis, there was no significant difference between the healthy and tumor‐bearing cohorts measured in the portal vein; however, the oxygen saturation of blood in the hepatic vein was significantly lower in the mice with tumors compared to the healthy cohort. The effect was not observed in the portal vein, which would indicate that the effect is not systemic, but is instead caused as the blood passes through the liver. It is a well‐known facet of cancer that malignant cells tend to assimilate glucose at a higher rate than their normal counterparts.^30^ The extent to which this increased metabolic burden is oxidative, however, remains unclear. It is known that cancer cells exhibit a high rate of glycolysis even in aerobic conditions (the Warburg effect),^31^ but conventional acceptance of this being the only metabolic pathway is now being challenged. Although it has been noted that tumor cell proliferation increases oxygen consumption by tumor tissues,^32^, ^33^ accumulating evidence suggests that the Warburg effect is only one aspect of cancer metabolism, which otherwise includes aerobic glycolysis, increased pentose phosphate pathway, increased macromolecule biosynthesis through redox homeostasis, and autophagy.^34^

This relationship between tumor oxygen use and supply places systemic oxygen delivery (e.g., through hyperbaric therapy^35^) as a parameter of potential interest for assessment in the clinic. This has been explored previously using invasive means,^36^ and the sparsity of noninvasive, quantitative means of measurement^37^ provides a clear rationale for our study. Moreover, it is known that tumors in the liver have an impact on blood flow, but their effect on oxygenation is less understood due to a lack of noninvasive measurement techniques.^38^

When performing susceptibility mapping in the brain, it is usual to acquire data using a 3D gradient echo sequence, the advantages over 2D sequences being cited as superior SNR, and that 2D acquisitions may introduce phase inconsistencies among adjacent slices.^28^ It is also noted, however, that 2D data are compatible with QSM; and it has been suggested in an SWI study of the liver that a 3D sequence may not be suitable for abdominal imaging due to the long acquisition times.^39^

There are a number of recent examples in which the QSM application being explored has necessitated calculating susceptibility maps from 2D gradient echo data. These are instances that require fast acquisitions, such as in the case of functional QSM^40^ or for use in patients who are unable to remain still for the duration of the scan.^41^ The latter is particularly prevalent as the most well‐developed application of QSM is to assess changes in focal iron deposition in the brains of Parkinson or Huntington disease sufferers. The data in this study were all acquired with a 2D gradient echo sequence because minimizing acquisition time reduced subject mortality, particularly in the tumor‐bearing mice.

The difference in ShvO_2_ between the mice with tumors and healthy mice could have clinical potential. Data in both cases were acquired under normoxic conditions. As such, acquisition, necessitated little more than a single standard T_2_*‐weighted scan. Tumor burden was not measured in this study as the disease model gave rise to a large number of small tumors that spread diffusely throughout the liver volume. This meant that they were extremely difficult to identify when small, and as they grew had a tendency to clump together, making it impossible to differentiate between tumours when the livers had been excised. Future experimental work could be to perform a longitudinal study to characterize the correlation between tumor burden and ShvO_2_. Once established, this would open the possibility of using QSM to noninvasively diagnose or monitor liver cancer, differentiate between benign and malignant lesions, or even to gauge the efficacy of treatment regimes.

One limitation of this study was the inability to measure the susceptibility of the hepatic artery. At the resolution of the imaging protocol used here, the diameter of the hepatic artery is of the order of a single voxel, so, measurements were undermined by partial volume effects that are known to result in inaccurate estimations of susceptibility.^1^ The ability to measure the susceptibility, and subsequently calculate the oxygen saturation of all three major hepatic vessels, would allow a more complete characterization of hepatic hemodynamics, as well as giving greater insight into hepatic oxygen metabolism, increasing the clinical usefulness of the technique. Hepatic arterial susceptibility measurements may be made possible by acquiring higher resolution images or by using larger animals (e.g., rats); however difficulty may be encountered due to pulsatile flow in the arterial vessel. By the same token, the measurement of susceptibility in the IVC on the QSM images was precluded by the proximity of the IVC to the hepatic artery. The acquisition sequence was not cardiac‐gated, so did not mitigate artefacts caused by the pulsatile nature of the blood flow in the hepatic artery. This resulted in an artefact along the phase encode direction of the image, acquired such that the artefact passes through the IVC.

The extraction of blood from the portal or hepatic veins of a mouse proved to be technically challenging due to its size, so the blood for invasive measurements was extracted from the IVC. While this is accepted by the authors as a limitation, it is noted that the liver contains 10% to 15% of the total blood volume,^42^ receives 25% of the cardiac output,^43^ and consumes ~20% of total resting oxygen.^44^ Furthermore, the hepatic veins are major contributors to the IVC, alongside smaller contributions from the renal and iliac veins. As such, it was assumed that the oxygenation of the blood in the IVC is a reasonable proxy for that in the hepatic veins.

From a clinical translation point of view, this experiment should be relatively easy to implement in humans. It has been shown in a recent study that QSM data from the entire liver can be acquired in ~19 sec, i.e. within one breath hold.^45^ Data could simply be acquired under normoxic conditions, but equally both patients and healthy volunteers tolerate hyperoxia well. Previous experiments examining the use of QSM in the liver^46^, ^47^ have focused on quantifying iron in the liver parenchyma, requiring complicated modifications of acquisition and processing protocols to account for fat. One major advantage of measuring the susceptibility of blood in the large vessels is that no fat is present; so data can be acquired and processed via standard means, although time‐consuming manual masking and ROI‐drawing were necessary. Equally, parenchymal iron will have no bearing on the measurements discussed in this experiment. The calculation of magnetic susceptibility from the phase image—a process also referred to in the literature as *inversion*—rectifies the nonlocal effects of susceptibility differences observed in phase images.^48^ The measurements made on QSM images reflect the properties of the underlying tissues, independent of orientation or the properties of neighboring tissues.^22^ In this instance, susceptibility measurements were taken from the blood vessels and water reference, i.e. areas that do not contain liver iron.

The use of an internal reference for measurements from QSM images can be problematic. In the (human) brain, variability has been shown in a number of regions commonly used as references in QSM studies. CSF has been shown to have the smallest variability (mean susceptibility 10 ± 14 ppb), whereas in the same study the mean susceptibility of the white matter ranged from 6 ± 20 ppb to 28 ± 23 ppb depending on the location in which it was measured.^19^ Furthermore, it has been shown that the susceptibility of CSF can change under the administration of gases.^2^ As such, standardization of the susceptibility reference is an important factor when trying to maximize the benefit of the quantitative nature of QSM. Performing QSM in the liver presents additional challenges for identifying a reference ROI. In comparison to the brain, the liver is an amorphous structure and is relatively homogeneous. As such, it is difficult to select an internal reference that is conspicuous enough not only to be identified repeatedly in the same animal but is clear and unambiguous in an entire study cohort. This difficulty is compounded by the fact that the anatomical position of the liver results in significant movement over the course of the respiratory cycle.

QSM facilitates the measurement of susceptibility variations based on the distortion of the B_0_ field within the FOV of the image. As such, it is not necessary that the reference be internal to the subject, just that it is independent of experimental variables, easy to depict and delineate, and easily identifiable across a wide range of subjects.^19^ The external reference used here meets each of these criteria; its inclusion in the experimental setup is straightforward, although one limitation is that the image FOV must be increased to accommodate it, potentially resulting in longer acquisition times if resolution is to be maintained.

The preliminary nature of this study means that there is some room for methodological improvement. For example, the control group selected for the tumor‐bearing cohort of CD1 severe combined immunodeficiency mice was nontumor‐bearing CD1 nonsevere combined immunodeficiency mice. Although a limitation of the study, it was decided that this would reduce the number and severity of the interventions performed on the animals. Our assumption in this case was that the blood flow to the liver would be unaffected between the groups. This is an issue that could be addressed in future studies.

Investigators were not blinded to the disease and healthy cohorts for this experiment. Data acquisition, processing, and analysis were all carried out by the same person. Obvious differences between the cohorts precluded the possibility of blinding. Although this could be included in the methodology in future studies, it would necessitate that all image processing (including masking and ROI segmentation) be performed by one party while another performed the experimental analysis. However, animals were selected and scanned at random from either cohort as an attempt to minimize unconscious bias.

The ability to noninvasively perform venous oximetry in the liver could have important clinical implications. Hepatic venous oxygen saturation is a useful metric and has been used to assess hepatic oxygen kinetics in studies focusing on topics as diverse as hemodialysis,^49^ acute and chronic heart failure,^50^ and hepatic ischemic/reperfusion injuries.^51^, ^52^ Furthermore, improvements in diagnostic radiology, patient selection, and operative technique mean that partial hepatectomy has increasingly become a more viable treatment option in cases of hepatic lesions, both malignant and benign. It is known that the regenerating liver places an increased metabolic burden on patients who have undergone the procedure and previously has been shown that ShvO_2_ reflects the metabolic status of the remnant liver.^11^, ^13^ The ability to relate magnetic susceptibility to SvO_2_ through QSM offers a way to assess this in a noninvasive fashion.

In this study, we employed QSM to assess changes in SvO_2_ in the portal and hepatic veins, which we modulated with a hyperoxic gas challenge. This is the first report that examines the ability of QSM to assess oxygen changes in the major hepatic vessels. We have shown that it is possible to detect statistically significant differences in blood oxygenation in response to hyperoxia. Moreover, our measurements showed good accordance with invasive measurements from the IVC made with a blood gas analyzer, and that it is possible to detect significant differences between the hepatic venous oxygen saturations of a group of healthy animals and a group with liver cancer.

## 5 CONCLUSION

QSM is a feasible tool for noninvasively measuring blood oxygenation in the major hepatic vessels. Measurements derived from QSM images can detect differences in ShvO_2_ between well‐oxygenated and partially deoxygenated blood, and furthermore can detect differences between the ShvO_2_ of healthy mice and mice with tumors.
